# New Base for Teeth

**Published:** 1875-05

**Authors:** 


### ARTICLE XV.

### *New Base for Teeth.*

Dr. W. C. Barrett, of Warsaw, N. Y., President of the Dental Society of the State of New York, has just shown us a new base for artificial teeth, which, while it resembles rubber in all desirable points, seems to have some advantages over that base. The inventor asserts that it may be colored to imitate the gums, without admixture of a compound of mercury. It is a wonderful substance, and if the statements made by the inventor are accurate, is likely to be largely used in the stead of rubber. It is produced by an inexpensive process, from the *Euphorbia Corollata*, or common milk-weed. The first set of teeth vulcanized on this base Dr. Barrett made himself, and exhibited to us. A meeting of well known members of the profession was called at the office of Dr. O. E. Hill, of Brooklyn, and several sets, one of them put up by Dr. Hill, were shown. Though the base is entirely new, and its properties imperfectly understood, and though no one of those who used it had any previous experience with it, every set attempted was a success, closely fitting the plaster mold. The base seemed to be even stronger than rubber. We are promised a description at length for our next issue.

The inventor asserts that it will relieve those who use it from liability under the claims of the Cummings Patent.—*Ed. in Dental Miscellany.*

---

